# Planned drug holidays during treatment with lenvatinib for radioiodine-refractory differentiated thyroid cancer: a retrospective study

**DOI:** 10.3389/fonc.2023.1139659

**Published:** 2023-10-11

**Authors:** Chihiro Matsuyama, Tomohiro Enokida, Yuri Ueda, Shinya Suzuki, Takao Fujisawa, Kazue Ito, Susumu Okano, Makoto Tahara

**Affiliations:** ^1^ Department of Pharmacy, National Cancer Center Hospital East, Kashiwa, Japan; ^2^ Department of Clinical Pharmacology and Therapeutics, Kyoto University Hospital, Kyoto, Japan; ^3^ Department of Head and Neck Medical Oncology, National Cancer Center Hospital East, Kashiwa, Japan; ^4^ Department of Otorhinolaryngology-Head and Neck Surgery, Tokyo Medical University, Shinjuku, Japan; ^5^ Department of Head and Neck Medical Oncology, Miyagi Cancer Center, Natori, Japan

**Keywords:** lenvatinib, thyroid cancer, oral anticancer agent, VEGF tyrosine kinase inhibitor, adverse events, planned drug holidays

## Abstract

**Background:**

In the phase 3 SELECT study, lenvatinib significantly improved prognostic outcomes vs. placebo in patients with radioiodine-refractory differentiated thyroid cancer (RR-DTC). However, toxicity of lenvatinib is sometimes considerable and requires frequent dose interruptions and modifications. Recently, planned drug holidays have been proposed as a means of avoiding severe adverse events (AEs).

**Methods:**

We retrospectively reviewed medical records to compare the efficacy and safety of lenvatinib in RR-DTC patients who underwent planned drug holidays (planned holiday group) vs. those who received conventional daily oral administration (daily group).

**Results:**

The subjects were 25 patients in the planned holiday group and 21 in the daily group. Median age was 73 years (range 43-84) and 62 years (range 42-75), and histologic subtype of papillary/follicular was 21/4 cases and 15/6 cases, respectively. Time to treatment failure (TTF) and overall survival (OS) were significantly longer in the planned holiday group than the daily group (not reached [NR] vs. 14.9 months, hazard ratio [HR] 0.25, 95% confidence interval [Cl] 0.11-0.58, p<0.001; NR vs. 26.6 months, HR 0.20, 95% CI 0.073-0.58, p=0.001, respectively). Median progression-free survival (PFS) was NR in the planned holiday group vs. 15.1 months in the daily group (HR 0.31, 95% CI 0.14-0.68, p=0.002). Duration of the period with lenvatinib dose ≥10 mg was significantly longer in the planned holiday group (NR vs. 6.5 months, HR 0.22, 95% CI 0.10-0.49, p<0.001), and the frequency of drug interruption due to intolerable AEs was lower (68.0% vs. 95.2%, p=0.027).

**Conclusion:**

Planned drug holidays for lenvatinib demonstrated significantly longer PFS, TTF, and OS than daily oral administration, and less intolerable toxicity leading to further unplanned treatment interruption. These benefits were apparently associated with a more extended period of lenvatinib administration at ≥10 mg. These findings might contribute to a favorable patient prognosis and safer toxicity profile.

## 1 Introduction

Thyroid cancer accounts for 1% of all cancer cases and 90% of malignant endocrine tumors. The prognosis of thyroid cancer is favorable, with a 10-year survival rate of 85%. Further, the incidence of distant metastasis is less than 5%, and 10-year survival in patients with metastasis is 25%–42% (1, 2). Currently, the initial treatment for differentiated thyroid cancer (DTC) is surgery, followed in some high-risk cases by radioactive iodine internal radiation iodine therapy (RAI) and TSH suppression using ^131^I (1). However, prognosis is poor in patients who are refractory to RAI (3). Lenvatinib is an oral multikinase inhibitor that inhibits vascular endothelial growth factor receptors (VEGFR) 1–3, fibroblast growth factor receptors (FGFR) 1–4, platelet-derived growth factor receptor a, and RET and KIT signaling pathways (4–6). In the SELECT trial, an international randomized phase 3 study in patients with RAI-refractory thyroid cancer, lenvatinib significantly prolonged median progression-free survival (PFS) to 18.3 months, compared to 3.6 months with a placebo, and improved overall response rate (ORR) (7). Accordingly, lenvatinib is now considered the standard therapy for thyroid cancer which is unresectable and refractory to treatment with radioactive iodine.

Despite these significant treatment benefits, the incidence of adverse events (AEs) in the SELECT trial was 97.3%, and 89.7% of patients experienced temporary interruption or dose reduction of lenvatinib (7). A subanalysis of the Japanese population also showed that all cases experienced AEs, and 93.3% of cases required temporary drug interruption or dose reductions to manage AEs due to lenvatinib (8). A systematic review reported that more than half of patients treated with lenvatinib experienced AEs such as proteinuria and fatigue, and 15%–25% of patients had grade > 3 AEs, including thrombocytopenia and hypertension (9). These findings indicate that continuation of lenvatinib requires proper management of adverse drug reactions. Another subgroup analysis of the SELECT study reported a negative correlation between treatment effect and duration of drug interruption due to lenvatinib-induced AEs. This study suggested that minimizing toxicity-induced continuous lenvatinib interruption might prolong PFS (10). Together, these findings indicate that improving treatment outcomes in lenvatinib therapy requires adequate management against AEs that may cause prolonged treatment interruption.

One general strategy in the management of intolerable AEs is rescheduling of the administration schedule. For example, the standard treatment schedule for sunitinib, regorafenib, and S-1 is four weeks of administration and two weeks of drug holiday. However, toxicities became more acceptable when the administration schedule was modified to two weeks on one-week off, without any sacrifice in anti-tumor effects (11–13). Regarding lenvatinib for hepatocellular carcinoma, weekend-off administration is reportedly helpful in maintaining therapeutic effect and improving overall survival (14). Moreover, planned drug holidays have been proposed to avoid severe AEs in thyroid cancer (15). The planned drug holiday is adjusted depending on the patient’s AE pattern, which means that the strategy is considered only for patients with intolerable AEs and is not constitutionally applied in patients who can tolerate daily administration. Thus, planned drug holidays appear a promising strategy for the continuation of anticancer treatment in patients intolerant of conventional schedules. However, the benefits of planned drug holidays have yet to be clarified.

Here, we report the potential impact of planned drug holiday administration on patient prognosis and safety in DTC patients, as well as compliance with lenvatinib.

## 2 Materials and methods

### 2.1 Patients

We retrospectively reviewed the medical records of recurrent or metastatic thyroid cancer patients treated with lenvatinib at the National Cancer Center Hospital East, Kashiwa, Japan, from May 2011 to December 2019 and compared the efficacy and safety of planned drug holiday administration with daily administration. Inclusion criteria were (1) pathologically proven papillary or follicular thyroid cancer, and (2) RAI refractory or RAI not indicated. Exclusion criteria were (1) histology of poorly differentiated cancer, medullary cancer, and anaplastic thyroid cancer, (2) indication for definitive treatment (surgery or radiotherapy), (3) every other day administration not defined as planned drug holiday administration and (4) daily protocol administration in a phase 2 or 3 clinical trial of a drug and subsequent planned drug holiday administration in daily practice after post-marketing in Japan. Written informed consent for Lenvatinib therapy was obtained from each patient. In the process, a potential modification of the treatment schedule and drug dose considering the adverse event was explained. The degree of proteinuria was assessed by the dipstick method in all cases throughout treatment (16), with grade 3 proteinuria defined as 3+ or above, as measured by the test in the current study. The study for summarizing their clinical information was approved by the Clinical Research and Ethical Review Board of the National Cancer Center East (task number: 2016-245).

### 2.2 Definition of planned drug holiday administration

**Figure 1:**
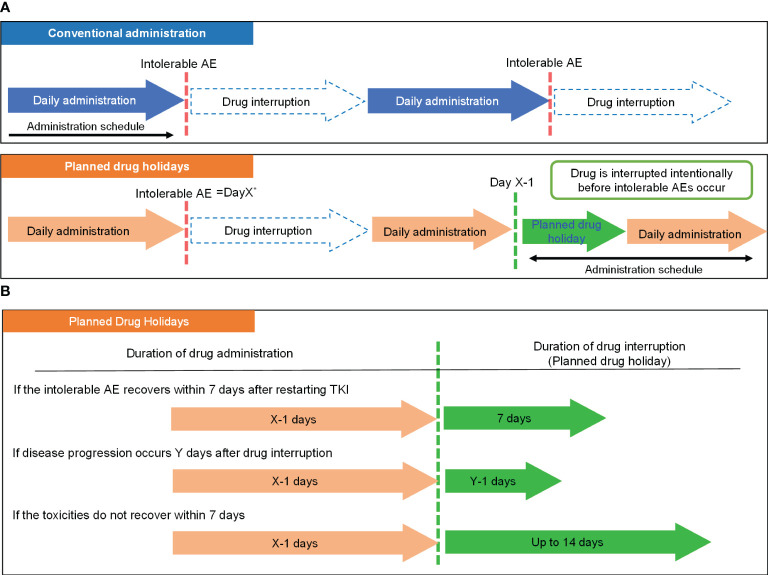
Concept of the planned drug holiday strategy. **(A)** Comparison of the basic strategy of conventional administration and planned drug holiday-based administration. **(B)** Determination of the duration of drug holiday by clinical situation. ^*^Day X: Day on which intolerable AEs occurred. ^**^Day Y: Day on which disease progression occurred. AE, Adverse Event; TKI, tyrosine kinase inhibitor.

The actual procedure of the drug holiday is demonstrated in **Figure 1**. We defined a planned drug holiday as an intentional drug interruption to avoid a repeat of treatment withdrawal which would eventually leads to tumor regrowth due to intolerable AEs. The schedule of the planned drug holiday was set as follows: if severe or intolerable AEs occurred at X days after the initiation of lenvatinib, administration in the next cycle should continue until day “X-1” (**Figure 1A**). Following initial introduction, the duration of drug interruption (duration of drug holiday) is determined according to recovery from the corresponding AEs, and the presence or absence of tumor progression during the treatment interruption. In patients whose AEs recovered within seven days after treatment cessation, a one-week drug holiday was applied. In patients whose tumor growth occurred Y days after treatment cessation and Y was less than one week, a drug holiday duration of “Y-1” days was applied. On the other hand, if a patient did not recover from the AE within one week after treatment cessation, the planned drug holiday could be extended to 14 days if disease progression did not occur (**Figure 1B**), and if an adverse event did not improve after 14 days off or day “Y-1”, dose reduction should be considered (15).

### 2.3 Evaluation of efficacy and statistical analysis

Clinical response to treatment was evaluated radiographically using computerized tomography or magnetic resonance imaging approximately every eight weeks until disease progression or treatment discontinuation. Anti-tumor activity was confirmed according to the Response Evaluation Criteria in Solid Tumors (RECIST) v.1.1 (17) by review of imaging results. After completing treatment, disease progression, survival status, and any further anticancer treatment were documented until death or loss to follow-up. All disease progression was determined radiologically using computed tomography or magnetic resonance imaging. The event of PFS was defined as disease progression or death from any cause, while the event of time to treatment failure (TTF) was determined as lenvatinib discontinuation or death from any cause. Namely, if lenvatinib was continued when disease progression occurred, given concerns about rapid tumor regrowth, it was defined as an event of PFS but not TTF. The event of overall survival (OS) was determined as death from any cause. Evaluation of duration was limited to doses of ≥10 mg/day since 10 mg was determined as a minimum dose in the SELECT trial (7).

Bivariate analyses were employed to examine differences in background characteristics, with the t-test used for continuous variables and the chi-square test or Fisher’s exact probability test for categorical variables. AE grade was evaluated according to CTCAE version 4.0. PFS, TTF, OS, and duration of dosing of ≥10 mg were calculated by the Kaplan-Meier product-limit method. The log-rank test was used to compare the survival or treatment duration of the two groups. All data were analyzed using SPSS version 22.0 (SPSS Inc., Chicago, IL), and p-values of <0.05 were considered statistically significant.

## 3 Results

### 3.1 Patient and treatment characteristics

Seventy recurrent or metastatic thyroid cancer patients treated with lenvatinib were available for review. The following cases were excluded: those with every other day administration (n=1), daily administration with a phase 2 or 3 trial followed by planned holiday administration after the trial (n=10), and those with anaplastic thyroid cancer (n=8) or medullary thyroid cancer (n=5). Finally, 46 patients were included in the analysis, 25 in the planned holiday group and 21 in the daily group. The patients in the daily group were treated between September 2011 and December 2017, while those in the planned holiday group were treated between March 2016 and June 2019, and the AEs, which led to the introduction of the planned holiday, are listed in **Supplementary Table 1**. Twenty-one patients had papillary thyroid cancer (PTC) and 4 had follicular thyroid cancer (FTC) in the planned holiday group, versus 15 and 6 patients in the daily group, respectively. More than 80% of patients received surgery or iodine-131 therapy before lenvatinib. Demographic and baseline characteristics were balanced between treatment arms, except for slightly younger age in the daily group (**Table 1**).

**Table 1:** Patient characteristics.

	Planned holiday group n=25	Daily group n=21	*p*-value^*^
Median age (years) [range]	73 [43-84]	62 [42-75]	0.020
Sex Male Female	7 (30.7%) 18 (69.3%)	6 (27.2%) 15 (72.8%)	1.000
ECOG performance status 0 1 2/3	14 (56.0%) 11 (44.0%) 0 (0%)	11 (52.3%) 10 (47.6%) 0 (0%)	0.806
Prior therapy Anticancer surgical therapy Iodine-131 therapy Anticancer chemotherapy	20 (80.0%) 20 (80.0%) 2 (8.0%)	20 (90.1%) 20 (90.1%) 4 (18.1%)	0.198 0.198 0.390
Histologic subtype Papillary carcinoma Follicular carcinoma	21 (84.0%) 4 (16.0%)	15 (68.1%) 6 (22.7%)	0.475
Metastatic lesions Lymph nodes Lung Bone Others	15 (60.0%) 19 (76.0%) 7 (28.0%) 3 (12.0%)	13 (63.6%) 17 (81.8%) 9 (40.9%) 3 (13.6%)	0.895 0.735 0.292 1.000

ECOG, Eastern Cooperative Oncology Group. ^*^P values were determined using the Mann-Whitney U test, χ^2^ test or Fisher’s exact probability test.

Details of treatment delivery by group are shown in **Table 2**. In the planned drug holiday group, median period from treatment initiation to the introduction of a planned drug holiday was 167 days (range 14-686) with the cumulative lenvatinib dose of 1746 mg (range 264-13412). The median lenvatinib dose at the point was 14 mg (range 10-24) daily, and the median administration and drug holiday period was eight days (range 4-21) and seven days (range 2-14), respectively. The total lenvatininb dose after the initiation of the planned drug holiday was 2641 mg (range 80-7000). During the study period, although the cumulative number of days of drug interruption was longer in the planned drug holiday group (260 days vs. 72 days, *p*<0.001), the eventual cumulative lenvatinib dose was substantially higher in that group than in the daily group (7458 vs. 3200, *p*=0.002).

**Table 2:** Median data of lenvatinib administration.

	Planned holiday group n=25	Daily group n=21	*p-*value^*^
Time from treatment initiation of planned holiday (days) [range]	167 [14-686]	N.A.	N.A.
Cumulative administered lenvatinib dose (mg) [range]	7458 [1596-21495]	3200 [696-22586]	0.002
Cumulative interruption during the overall period (days) [range]	260 [4-652]	72 [0-217]	<0.001
Cumulative unexpected interruption during the overall period (days) [range]	6 [6-523]	72 [0-217]	0.683
Cumulative unexpected interruption during the planned drug holiday administration period(days) [range]	1 [0-59]		<0.001
Dose intensity^‡^ during the overall period (days) [range]	0.87 [0.44-0.99]	0.75 [0.47-1.00]	0.032
Dose intensity^‡^ during the planned drug holiday administration period (days) [range]	0.99 [0.77-1.00]		<0.001
Duration of unexpected drug interruption per interruption (days) [range]	0 [0-42]	12 [0-181]	<0.001

N.A., not available. ^*^P values were determined using the Mann-Whitney U test, χ^2^ test or Fisher’s exact probability test. ^‡^Ratio of administered cumulative dose (mg) to planned cumulative dose (mg).

### 3.2 Impact on clinical response, treatment duration, and survival

**Figure 2:**
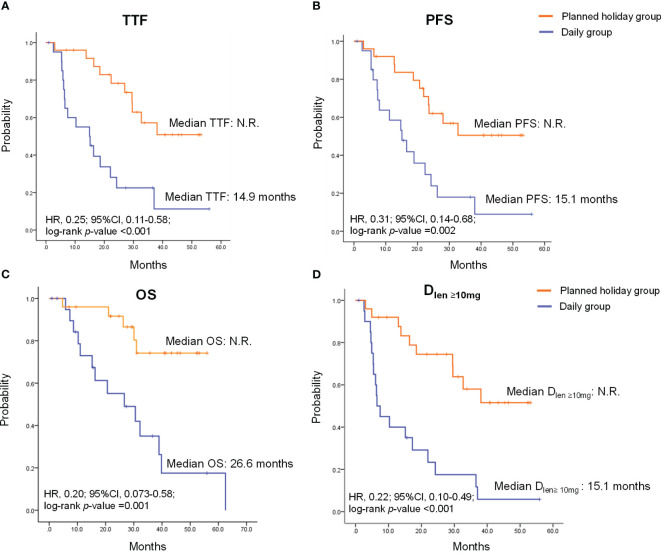
Time to treatment failure (TTF), progression-free survival (PFS), overall survival (OS) and duration of the period with lenvatinib dose ≥10 mg/day (D_len_ ≥10mg) in patients treated with lenvatinib. **(A)** TTF. **(B)** PFS. **(C)** OS **(D)** D_len_ ≥10mg. HR, hazard ratio; CI, confidence interval; N.R., not reached.

Overall response rate (ORR) was 62.5% in all patients and did not statistically differ between the planned drug holiday and daily groups (65.0% vs. 60.0%, *p*=1.000) (**Table 3**). TTF and PFS were significantly longer in the planned holiday group than the daily group (TTF: not reached [N.R.] vs. 14.9 months, hazard ratio [HR] 0.25, 95% confidence interval [Cl] 0.11-0.58, *p*<0.001. PFS: N.R. vs. 15.1 months, HR 0.31, 95% CI 0.14-0.68, *p*=0.002). Further, OS was significantly longer in the planned holiday group than in the daily group (N.R. vs. 26.6 months, HR, 0.20; 95% CI, 0.073-0.58; *p*=0.001). Duration of the period with lenvatinib dose ≥10 mg/day was significantly longer in the planned holiday group (N.R. vs. 6.5 months, HR 0.22; 95% CI 0.10-0.49, *p*<0.001) (**Figure 2**).

**Table 3:** Overall response rate.

	All patients N=46 (%)	Planned holiday group n=25 (%)	Daily group n=21 (%)	*p*-value^*^
Complete response (CR)	0 (0)	0 (0)	0 (0)	N.A.
Partial response (PR)	25 (54.3)	13 (52.0)	12 (57.1)	0.959
Stable disease (SD)	13 (28.2)	5 (20.0)	8 (38.0)	0.303
Progressive disease (PD)	2 (4.3)	2 (8.0)	0 (0)	0.493
Could not be evaluated	6 (13.0)	5 (20.0)	1 (4.7)	0.198
Overall response rate (ORR)^†^	62.5%	65.0%	60.0%	1.000

N.A., not available. ^*^P values were determined using the χ2 test or Fisher’s exact probability test. ^†^ORR was calculated in those patients who could be evaluated radiographically.

### 3.3 Impact on adverse events

The observed AEs, and reasons for unplanned drug interruption and the introduction of planned drug holidays are presented in **Tables 4** and **5**, respectively. Significant differences in the incidence of AEs between the planned drug holiday and daily group were as follows: grade 1 or 2 nausea was frequent in the daily group (0% vs. 33.3%, p=0.002), while grade 3 proteinuria was more frequent in the planned drug holiday group (60.0% vs. 23.8%, p<0.01) (**Table 4**). Fewer patients required unplanned drug interruption due to AEs in the planned holiday group (68.0% vs. 95.0%; *p*=0.027) (**Table 5**). During the whole treatment period, unplanned drug interruptions per patient was significantly lower in the planned holiday group (1.4 times/patient vs. 6.7 times/patient, *p*<0.001). Among AEs which caused unplanned drug interruption, fatigue/malaise (4.0% vs. 52.3%, *p*<0.001), diarrhea (8.0% vs. 38.0%, *p*=0.028), and thrombocytopenia (0% vs. 19.0%, *p*=0.037) were statistically less frequent in the planned holiday group (**Table 5**).

**Table 4:** Adverse Events due to Lenvatinib.

AE	Grade 1 or 2			Grade 3 or 4		
	Planned holiday group n=25 n (%)	Daily group n=21 n (%)	*p*-value^*^	Planned holiday group n=25 n (%)	Daily group n=21 n (%)	*p*-value^*^
Any AE	25 (100)	21 (100)	N.A.	15 (60.0)	7 (33.3)	0.071
Hypertension	23 (92.0)	20 (95.2)	1.000	6 (24.0)	7 (33.3)	0.484
Proteinuria	22 (88.0)	16 (76.1)	0.439	15 (60.0)	5 (23.8)	0.014
PPEs	21 (84.0)	12 (57.1)	0.092	1 (4.0)	0 (0)	1.000
Hypoalbuminemia	21 (84.0)	20 (95.2)	0.357	0 (0)	0 (0)	N.A.
Fatigue/malaise	18 (72.0)	12 (57.1)	0.292	0 (0)	2 (9.5)	0.203
Anorexia	14 (56.0)	13 (61.9)	0.685	1 (4.0)	2 (9.5)	0.585
Peripheral edema	14 (56.0)	11 (52.3)	0.806	0 (0)	0 (0)	N.A.
Hypertriglyceridemia	12 (48.0)	13 (61.9)	0.234	1 (4.0)	0 (0)	1.000
Creatinine increased	11 (44.0)	6 (28.5)	0.418	0 (0)	0 (0)	N.A.
Anemia	9 (36.0)	4 (19.0)	0.203	0 (0)	0 (0)	N.A.
Hypercholesterolemia	8 (32.0)	12 (57.1)	0.087	0 (0)	1 (4.7)	0.457
Diarrhea	7 (28.0)	11 (52.3)	0.091	0 (0)	1 (4.7)	0.457
AST increased	7 (28.0)	8 (38.0)	0.467	2 (8.0)	2 (9.5)	1.000
ALT increased	6 (24.0)	8 (38.0)	0.301	2 (8.0)	2 (9.5)	1.000
Thrombocytopenia	6 (24.0)	10 (47.6)	0.094	0 (0)	2 (9.5)	0.203
Hemorrhage	1 (4.0)	1 (4.7)	1.000	0 (0)	0 (0)	N.A.
Nausea	0 (0)	7 (33.3)	0.002	0 (0)	1 (4.7)	0.457

N.A., not available; AE, adverse event; ALT, alanine aminotransferase; AST, aspartate aminotransferase; PPEs, palmar-plantar erythrodysesthesia syndrome. ^*^P values were determined using the χ2 test or Fisher’s exact probability test.

**Table 5:** Reason for unplanned drug interruption.

AE	Planned holiday group n=25 n (%)	Daily group n=21 n (%)	*p*-value^*^
Hypertension	1 (4.0)	3 (14.2)	0.318
Proteinuria	6 (24.0)	6 (28.5)	0.725
PPEs	7 (28.0)	7 (33.3)	0.695
Fatigue/malaise	1 (4.0)	11 (52.3)	<0.001
Anorexia	1 (4.0)	5 (23.8)	0.079
Peripheral edema	1 (4.0)	4 (19.0)	0.163
Anemia	1 (4.0)	0 (0)	1.000
Diarrhea	2 (8.0)	8 (38.0)	0.028
AST increased	0 (0)	0 (0)	N.A.
ALT increased	0 (0)	0 (0)	N.A.
Thrombocytopenia	0 (0)	4 (19.0)	0.037
Nausea	0 (0)	3 (14.2)	0.088
Vomiting	0 (0)	1 (4.7)	0.457
Other AE^†^	0 (0)	0 (0)	N.A.
Patients who required an unexpected drug interruption (%)	17 (68.0)	20 (95.2)	0.027
Number of unexpected drug interruptions per patient (times/patient)	1.4	6.7	<0.001

N.A., not available; AE, adverse event; ALT, alanine aminotransferase; AST, aspartate aminotransferase; PPEs, palmar-plantar erythrodysesthesia syndrome. ^*^P values were determined using the χ2 test or Fisher’s exact probability test. ^†^Other AEs: hoarseness (n=1, 4%), anal pain (n=1, 4%), and arthralgia (n=1, 4%).

## 4 Discussion

This study suggests that planned drug holiday administration of lenvatinib has significant benefits in survival and safety profile in patients with thyroid cancer. This strategy improved patient prognosis and had a better toxicity profile, probably through the avoidance of undesirable drug withdrawal and the relatively long duration of period with a higher (≥10 mg) dose of lenvatinib per single day through prevention of the occurrence of severe AEs.

Severe AEs often require prolonged drug withdrawal and frequent dose modification. In this situation, the planned drug holiday strategy enables oncologists to adjust the lenvatinib schedule based on the status of patient AEs. Interruption before the development of severe AEs can help avoid extended drug withdrawal and continue treatment (15). Similarly, administration with a scheduled drug holiday was reportedly associated with fewer toxicities and improved survival benefits in patients treated with VEGFR tyrosine kinase inhibitors such as sunitinib for renal cell cancer and lenvatinib for hepatocellular cancer (11, 14). These findings have led to speculation that a shorter treatment cycle may lead to a lower incidence of AEs, and associated longer drug exposure. Accordingly, the duration of unplanned drug interruption was shorter with fewer grade 3 or 4 AEs, except for proteinuria, which is more likely to occur with prolonged drug use (18), and ultimately to longer treatment duration in the planned holiday group than the daily group. Furthermore, we believe that our strategy, which modifies the treatment schedule according to the individual actual occurrence of AEs, would be more minute on maximizing therapeutic efficacy by maintaining the period of lenvatinib administration with safe, compared with the fixed or mandatory drug holidays strategies (i.e., two-weeks-on/one-week-off, weekends-off) (11, 14).

One concern with drug holidays relates to tumor growth during the planned drug interruption. The duration of our planned drug holiday was therefore determined with regard to not only AE status but also tumor progression; if disease progression occurred Y days after drug interruption, day “Y-1” was suggested as the planned drug holiday duration, with the result that the median drug holiday duration was 7 days. Considering that Yamazaki et al. reported a median time from lenvatinib cessation to tumor progression of nine days in eight thyroid cancer patients who experienced rapid tumor progression (flare phenomenon) after discontinuation of Lenvatinib (19), this length of treatment interruption (seven days) appears reasonable.

A further question is the inverse relationship between drug interruption and PFS observed in the SELECT study, in which a duration of lenvatinib interruption of more than 10% of the overall treatment period was associated with a significantly shorter PFS than an interruption of less than 10%. In contrast to that finding, despite a longer cumulative duration of dose interruption in the drug holiday, PFS was significantly longer than in the daily group. We believe that this difference can be explained by the longer duration of a relatively high dose (herein >10mg/day) of lenvatinib in the drug holiday group. Supporting this assumption, the previous reports indicated the importance of a daily dose of lenvatinib through their experience of “rechallenge” with a higher dose of lenvatinib after progression at a lower dose. Considering that they usually adopted drug holidays when rechallenge, our strategy shares the exact same basis as these earlier reports while representing a more preemptive strategy that can contribute to a better overall safety profile. Although our single-center retrospective design is a likely limitation of our study, we believe that this strategy is worth prospective evaluation, in which an ethical review board-approved standardized drug holiday strategy should be applied and tested.

## 5 Conclusion

In thyroid cancer patients, the planned holiday demonstrated significantly longer PFS, TTF, and OS and less severe toxicity than daily administration, presumably due to safer lenvatinib administration with minimum treatment interruption as well as maintenance of the duration of a higher dose of daily lenvatinib.

## Data availability statement

The original contributions presented in the study are included in the article/**Supplementary Material**. Further inquiries can be directed to the corresponding author.

## Ethics statement

The studies involving human participants were reviewed and approved by Clinical Research and Ethical Review Board of the National Cancer Center East (task number: 2016-245). The patients/participants provided their written informed consent to participate in this study.

## Author contributions

CM, TE, YU, SS and MT participated in the study concept and design, interpreted the data, and drafted the manuscript. KI, TF, TE and SO extracted, managed and analyzed the data. All authors provided critical revisions and approved the final manuscript.

## References

[1] Chougnet C Brassard M Leboulleux S Baudin E Schlumberger M. Molecular targeted therapies for patients with refractory thyroid cancer. Clin Oncol (R Coll Radiol) (2010) 22(6):448–55. doi: 10.1016/j.clon.2010.04.008 20554167

[2] Durante C Haddy N Baudin E Leboulleux S Hartl D Travagli JP. Long-term outcome of 444 patients with distant metastases from papillary and follicular thyroid carcinoma: benefits and limits of radioiodine therapy. J Clin Endocrinol Metab (2006) 91(8):2892–9. doi: 10.1210/jc.2005-2838 16684830

[3] Pacini F Ito Y Luster M Pitoia F Robinson B Wirth L. Radioactive iodine-refractory differentiated thyroid cancer: unmet needs and future directions. Expert Rev Endocrinol Metab (2012) 7(5):541–54. doi: 10.1586/eem.12.36 30780891

[4] Matsui J Funahashi Y Uenaka T Watanabe T Tsuruoka A Asada M. Multi-kinase inhibitor E7080 suppresses lymph node and lung metastases of human mammary breast tumor MDA-MB-231 via inhibition of vascular endothelial growth factor-receptor (VEGF-R) 2 and VEGF-R3 kinase. Clin Cancer Res (2008) 14(17):5459–65. doi: 10.1158/1078-0432.CCR-07-5270 18765537

[5] Matsui J Yamamoto Y Funahashi Y Tsuruoka A Watanabe T Wakabayashi T. E7080, a novel inhibitor that targets multiple kinases, has potent antitumor activities against stem cell factor producing human small cell lung cancer H146, based on angiogenesis inhibition. Int J Cancer (2008) 122(3):664–71. doi: 10.1002/ijc.23131 17943726

[6] Okamoto K Kodama K Takase K Sugi NH Yamamoto Y Iwata M. Antitumor activities of the targeted multi-tyrosine kinase inhibitor lenvatinib (E7080) against RET gene fusion-driven tumor models. Cancer Lett (2013) 340(1):97–103. doi: 10.1016/j.canlet.2013.07.007 23856031

[7] Schlumberger M Tahara M Wirth LJ Robinson B Brose MS Elisei R. Lenvatinib versus placebo in radioiodine-refractory thyroid cancer. New Engl J Med (2015) 372(7):621–30. doi: 10.1056/NEJMoa1406470 25671254

[8] Kiyota N Schlumberger M Muro K Ando Y Takahashi S Kawai Y. Subgroup analysis of Japanese patients in a phase 3 study of lenvatinib in radioiodine-refractory differentiated thyroid cancer. Cancer Sci (2015) 106(12):1714–21. doi: 10.1111/cas.12826 PMC4714672 26426092

[9] Zhu C Ma X Hu Y Guo L Chen B Shen K. Safety and efficacy profile of lenvatinib in cancer therapy: a systematic review and meta-analysis. Oncotarget (2016) 7(28):44545–57. doi: 10.18632/oncotarget.10019 PMC5190117 27329593

[10] Tahara M Brose MS Wirth LJ Suzuki T Miyagishi H Fujino K. Impact of dose interruption on the efficacy of lenvatinib in a phase 3 study in patients with radioiodine-refractory differentiated thyroid cancer. Eur J Cancer (Oxford Engl 1990) (2019) 106:61–8. doi: 10.1016/j.ejca.2018.10.002 30471649

[11] Makino K Yoda K Tomoishi J Kume H. Efficacy and tolerability of a low-dose, 2-week administration of sunitinib followed by a week rest (2/1 schedule) for metastatic renal cell carcinoma: a single center experience of six cases. BMC Res Notes (2014) 7:872. doi: 10.1186/1756-0500-7-872 25471941 PMC4289163

[12] Yamatsuji T Fujiwara Y Matsumoto H Hato S Namikawa T Hanazaki K. Feasibility of oral administration of S-1 as adjuvant chemotherapy in gastric cancer: 4-week S-1 administration followed by 2-week rest vs. 2-week administration followed by 1-week rest. Mol Clin Oncol (2015) 3(3):527–32. doi: 10.3892/mco.2015.500 PMC4471633 26137261

[13] Petrioli R Chirra M Messuti L Fiaschi AI Savelli V Martellucci I. Efficacy and safety of regorafenib with 2/1 schedule for patients ≥ 75 years with metastatic colorectal cancer (mCRC) after failure of 2 lines of chemotherapy. Clin Colorectal Cancer (2018) 17(4):307–12. doi: 10.1016/j.clcc.2018.02.005 29548772

[14] Iwamoto H Suzuki H Shimose S Niizeki T Nakano M Shirono T. Weekends-off lenvatinib for unresectable hepatocellular carcinoma improves therapeutic response and tolerability toward adverse events. Cancers (Basel) (2020) 12(4):1010. doi: 10.3390/cancers12041010 32325921 PMC7226076

[15] Tahara M. Management of recurrent or metastatic thyroid cancer. ESMO Open (2018) 3(Suppl 1):e000359. doi: 10.1136/esmoopen-2018-000359 29713501 PMC5922569

[16] Iseki K Ikemiya Y Iseki C Takishita S. Proteinuria and the risk of developing end-stage renal disease. Kidney Int (2003) 63(4):1468–74. doi: 10.1046/j.1523-1755.2003.00868.x 12631363

[17] Eisenhauer EA Therasse P Bogaerts J LH S Sargent D Ford R. New response evaluation criteria in solid tumours: revised RECIST guideline (version 1.1). Eur J Cancer (Oxford Engl 1990) (2009) 45(2):228–47. doi: 10.1016/j.ejca.2008.10.026 19097774

[18] Iwasaki H Yamazaki H Takasaki H Suganuma N Sakai R Nakayama H. Renal dysfunction in patients with radioactive iodine-refractory thyroid cancer treated with tyrosine kinase inhibitors: A retrospective study. Med (Baltimore) (2019) 98(42):e17588–e. doi: 10.1097/MD.0000000000017588 PMC6824644 31626129

[19] Yamazaki H Sugino K Matsuzu K Masaki C Akaishi J Hames K. Rapid disease progression after discontinuation of lenvatinib in thyroid cancer. Med (Baltimore) (2020) 99(11):e19408. doi: 10.1097/MD.0000000000019408 PMC7220477 32176066

