# Supplementary material for: Planned drug holidays during treatment with lenvatinib for radioiodine-refractory differentiated thyroid cancer: a retrospective study
Source: Front Oncol. 2023 Oct 11;13:1139659. doi: 10.3389/fonc.2023.1139659 (PMC10598871; doi:10.3389/fonc.2023.1139659)
Supplement: Supplementary file 1 [file Table_1.docx]

**Supplementary Table 1. AEs leading to the Introduction of Planned drug holidays**

| Case | AE leading to the introduction of  the planned drug holidays | Case | AE leading to the introduction of  the planned drug holidays |
| --- | --- | --- | --- |
| #1 | Anorexia (Gr2) | #14 | PPEs (Gr2) |
| #2 | Proteinurea (Gr3) | #15 | AST increased (Gr1), ALT increased (Gr1) |
| #3 | Anorexia, Thrombocytopenia (Gr2) | #16 | Thrombocytopenia (Gr2),  Protein urea (Gr2), Hypertension (Gr2) |
| #4 | Fatigue/malaise (Gr2), Hypertension (Gr2) | #17 | Proteinurea (Gr3) |
| #5 | Fatigue/malaise (Gr2), Hypertension (Gr3) | #18 | Anorexia (Gr3), Proteinurea (Gr3) |
| #6 | Proteinurea (Gr3) | #19 | Anorexia (Gr2) |
| #7 | Fatigue/malaise (Gr1), Anorexia (Gr1), Thrombocytopenia (Gr1), PPEs (Gr1) | #20 | PPEs (Gr2) |
| #8 | PPEs (Gr2) | #21 | Anorexia (Gr2) |
| #9 | Thrombocytopenia (Gr2) | #22 | PPEs (Gr2) |
| #10 | Fatigue/malaise (Gr2) | #23 | Proteinurea (Gr3) |
| #11 | PPEs (Gr2) | #24 | PPEs (Gr2) |
| #12 | Fatigue/malaise (Gr1), Anorexia (Gr1),  PPEs (Gr2) | #25 | PPEs (Gr2) |
| #13 | Anal pain (Gr2) |  |  |

AE: adverse event, ALT: alanine aminotransferase, AST: aspartate aminotransferase, PPEs: palmar-plantar erythrodysesthesia syndrome.
